# ‘Value-based methodology for person-centred, integrated care supported by Information and Communication Technologies’ (ValueCare) for older people in Europe: study protocol for a pre-post controlled trial

**DOI:** 10.1186/s12877-022-03333-8

**Published:** 2022-08-17

**Authors:** E. L. S. Bally, A. van Grieken, L. Ye, M. Ferrando, M. Fernández-Salido, R. Dix, O. Zanutto, M. Gallucci, V. Vasiljev, A. Carroll, A. Darley, A. Gil-Salmerón, S. Ortet, T. Rentoumis, N. Kavoulis, O. Mayora-Ibarra, N. Karanasiou, G. Koutalieris, J. A. Hazelzet, B. Roozenbeek, D. W. J. Dippel, H. Raat, on behalf of the ValueCare consortium, Dorien Brouwer, Francesco Mattace-Raso, Demi Cheng, Mireia Ferri, Tamara Alhambra-Borrás, Jorge Garcés-Ferrer, Beatriz Vallina Acha, Elena Rocher, Stefania Macchione, Elena Procaccini, Tomislav Rukavina, Patrick Harnett, Natália Machado, Diana Guardado, Ana Filipa Leandro, Michele Marchesoni, Claudio Eccher, Sara Testa, Ioannis Arkoumanis, Athanasios Koumparos, Nhu Tram, Leo Lewis, Pilar Gangas Peiró, Natalia Allegretti, Karolina Mackiewicz

**Affiliations:** 1 Department of Public Health, Erasmus University Medical Center, Rotterdam, The Netherlands; 2 R&D+I Consultancy, Kveloce I+D+i (Senior Europa SL), Valencia, Spain; 3 Polibienestar Research Institute, University of Valencia, Valencia, Spain; 4 Fundación de La Comunidad Valenciana Para La Promoción Estratégica, El Desarrollo Y La Innovación Urbana (Las Naves), Valencia, Spain; 5 European Project Office Department, Istituto Per Servizi Di Ricovero E Assistenza Agli Anziani (Institute for Hospitalization and Care for the Elderly), Treviso, Italy; 6 Local Health Authority N.2 Treviso, Centre for Cognitive Disease and Dementia, Treviso, Italy; 7 Faculty of Medicine, Department of Social Medcine and Epidemiology, University of Rijeka, Rijeka, Croatia; 8 School of Medicine, University College Dublin, Dublin, Ireland; 9 International Foundation of Integrated Care, Oxford, UK; 10 Innovation Department, Cáritas Diocesana de Coimbra, Coimbra, Portugal; 11 Alliance for Integrated Care, Athens, Greece; 12 Athens Medical Center, Athens, Greece; 13 Center for Health and Wellbeing, Fondazione Bruno Kessler, Trento, Italy; 14 VIDAVO, Thessaloniki, Greece; 15 Vodafone Innovus, Athens, Greece; 16 Department of Neurology, Erasmus University Medical Center, Rotterdam, The Netherlands

**Keywords:** Integrated health and social care, Value-based health care, Patient-reported outcome measures, Older people, Pre-post controlled clinical trial, ICT support

## Abstract

**Background:**

Older people receive care from multiple providers which often results in a lack of coordination. The Information and Communication Technology (ICT) enabled value-based methodology for integrated care (ValueCare) project aims to develop and implement efficient outcome-based, integrated health and social care for older people with multimorbidity, and/or frailty, and/or mild to moderate cognitive impairment in seven sites (Athens, Greece; Coimbra, Portugal; Cork/Kerry, Ireland; Rijeka, Croatia; Rotterdam, the Netherlands; Treviso, Italy; and Valencia, Spain). We will evaluate the implementation and the outcomes of the ValueCare approach. This paper presents the study protocol of the ValueCare project; a protocol for a pre-post controlled study in seven large-scale sites in Europe over the period between 2021 and 2023.

**Methods:**

A pre-post controlled study design including three time points (baseline, post-intervention after 12 months, and follow-up after 18 months) and two groups (intervention and control group) will be utilised. In each site, (net) 240 older people (120 in the intervention group and 120 in the control group), 50–70 informal caregivers (e.g. relatives, friends), and 30–40 health and social care practitioners will be invited to participate and provide informed consent. Self-reported outcomes will be measured in multiple domains; for older people: health, wellbeing, quality of life, lifestyle behaviour, and health and social care use; for informal caregivers and health and social care practitioners: wellbeing, perceived burden and (job) satisfaction. In addition, implementation outcomes will be measured in terms of acceptability, appropriateness, feasibility, fidelity, and costs. To evaluate differences in outcomes between the intervention and control group (multilevel) logistic and linear regression analyses will be used. Qualitative analysis will be performed on the focus group data.

**Discussion:**

This study will provide new insights into the feasibility and effectiveness of a value-based methodology for integrated care supported by ICT for older people, their informal caregivers, and health and social care practitioners in seven different European settings.

**Trial registration:**

ISRCTN registry number is 25089186. Date of trial registration is 16/11/2021.

## Background

The increase in life expectancy observed globally is one of the greatest public health successes of the 20th Century. In 2019, the global population aged ≥ 65 years was estimated to be 703 million and this number is expected to double by 2050 [1]. Ageing is correlated with a higher risk of multimorbidity, frailty, and cognitive impairment [2–4]. Firstly, having two or more medical conditions and/or disabilities at the same time (i.e. multimorbidity) is increasingly common among older adults, as mortality rates have declined and the population has aged [4, 5]. Secondly, community-dwelling older adults are prone to developing frailty whereby multiple physiological systems gradually lose their intrinsic capacity [6, 7], which increases the risk of falls, disability, and long-term care [8, 9]. Finally, age-related diseases accelerate the decline in performance on cognitive abilities such as remembering, reasoning, and planning which can lead to the development of cognitive impairments [10].

Multimorbidity, frailty, and cognitive impairment can have significant implications for an older person’s functional independence and quality of life [10–12]. Furthermore, these conditions are correlated with an increased risk of unplanned health and care utilisation, especially costly hospital admissions, being thus challenging for the health and care systems related costs [11, 13, 14]. The objective, therefore, is to help maintaining older people’s intrinsic capacity and independence for as long as possible and to prevent hospitalisation. Integration of care will enable a proactive, predictive, and personalised delivery of health and social care and support services for this ageing population.

“Integration” of service delivery includes processes of linking and coordinating services to overcome fragmentation [15]. Older people receive care from multiple providers at various sites — outpatient units, primary care practices, specialty clinics, hospitals, and others – which often results in a lack of coordination. Integrated care aims to better articulate health and social care around the individual’s needs and therefore improve their health outcomes and experiences [16, 17]. Moreover, a recent meta-analysis has shown that integrated care is likely to reduce costs and to improve outcomes [18] such as reducing the risk of hospital admissions and increasing the patients’ care satisfaction [19–21]. In this regard, integrated care partnerships are increasingly acknowledged as an organising framework and mechanism to deliver value-based health care with the purpose of maximizing value for patients, health and care practitioners, managers, and policymakers [22].

Value can be defined as health outcomes achieved, relative to the costs of delivering these outcomes [23]. In a value-based system, outcomes are measured across the continuum of care and according to what is meaningful to its end users, such as functional status and quality of life [24, 25]. Standardisation of outcome measures is essential for improving care and supporting people living with a condition in making informed decisions with their care team members and service funders. This requires a combined effort by care team members in the continuum of care to collect data and to use data accordingly [26]. Furthermore, it requires Information and Communication Technologies (ICT) platforms that facilitate data sharing and support healthcare delivery [22, 26].

Whilst there is evidence showing the value of integrated care programs for older people [21, 27], previous research on ICT-enhanced integrated care interventions showed mixed results for this population. In this regard, Kim et al. (2021) found significant effects of ICT-enhanced integrated care management for frail older adults on overall quality of life and functional outcomes [28]. In contrast, studies by Mateo-Abdad et al. (2020) and Piera-Jiménez et al. (2020) reported that ICT-enhanced integrated care programs have only small clinical effects [29, 30]. There is a need for more knowledge on adapting ICT-enhanced integrated care interventions for older people to individual settings, the effectiveness of interventions in key target groups, and its cost-effectiveness [31, 32]. The purpose of this article is to describe the framework of the evaluation of the value-based methodology for integrated care supported by ICT developed by the ValueCare consortium members.

### Project ValueCare

ValueCare aims to deliver technology-enabled, outcome-based integrated health and social care for older people facing multimorbidity, and/or frailty, and/or mild to moderate cognitive impairment to improve their quality of life, thus supporting the sustainability of European health and social care systems. The ValueCare project is funded under the Horizon 2020 Topic call Digital Transformation in Health and Care, under Grant Agreement No. 875215. ValueCare is developing a robust, secure, and scalable digital solution which is co-designed with end users (older people, their informal caregivers, and health and social care practitioners). To this end, ValueCare aims to satisfy the ‘Quadruple Aim’ of improved care experience, better outcomes for citizens, optimisation in the use of resources, and job satisfaction and wellbeing of care team members [33]. In this project, seven large-scale sites in Europe (Athens, Greece; Coimbra, Portugal; Cork/Kerry, Ireland; Rijeka, Croatia; Rotterdam, the Netherlands; Treviso, Italy; and Valencia, Spain) will contribute to the implementation of the ValueCare approach in which each site is expected to adapt the general value-based methodology to their local context.

### Objectives

The aim of the study is to evaluate the ValueCare approach, using a pre-post controlled design, measuring the benefits for each one of the target groups (older people using health and social care services, their informal caregivers, and health and social care practitioners), and thus to be able to properly evaluate implementation outcomes. The specific objectives are:

1. To compare the benefits of the ValueCare approach versus usual care for older people with regard to indicators of health, wellbeing, quality of life, lifestyle behaviour, and health and social care use.
2. To evaluate the benefits of the ValueCare approach for older people’s caregivers (e.g. relatives, friends), and health and social care practitioners in terms of wellbeing, perceived burden and (job) satisfaction.
3. To evaluate the acceptability, appropriateness, feasibility, fidelity, and costs of the ValueCare approach.

### Hypotheses

Our hypothesis is that older people in the intervention group (i.e. individuals benefiting from ValueCare) have more favourable results with regard to indicators of health, wellbeing, quality of life, lifestyle behaviour, and reduced health and social care usage compared with older people participating in the control group (i.e. individuals receiving ‘usual care’). With respect to informal caregivers and health and social care practitioners, we expect a lower caregiver burden, and improved wellbeing and (job) satisfaction among participants in the intervention group. Furthermore, we hypothesise the costs of care for the intervention group will be lower, compared to the control group.

## Methods/design

### Study design

The evaluation of ValueCare has a pre-post controlled design with an intervention group (using the ‘ValueCare approach’) and a control group (‘care as usual’). Measurements are taken at baseline (*T*_0_), after 12 months (*T*_1_; the end of the ‘ValueCare approach’ intervention period), and at 18 months (*T*_2_) [32, 34, 35]. In each of the seven European countries, intervention and control sites (GP practices, community centres for health and wellbeing, and hospitals) are chosen. Table 1 shows the timeline of enrolment, interventions and assessments for this study. Baseline data collection is scheduled to commence by the end of 2021.

**Table 1:**
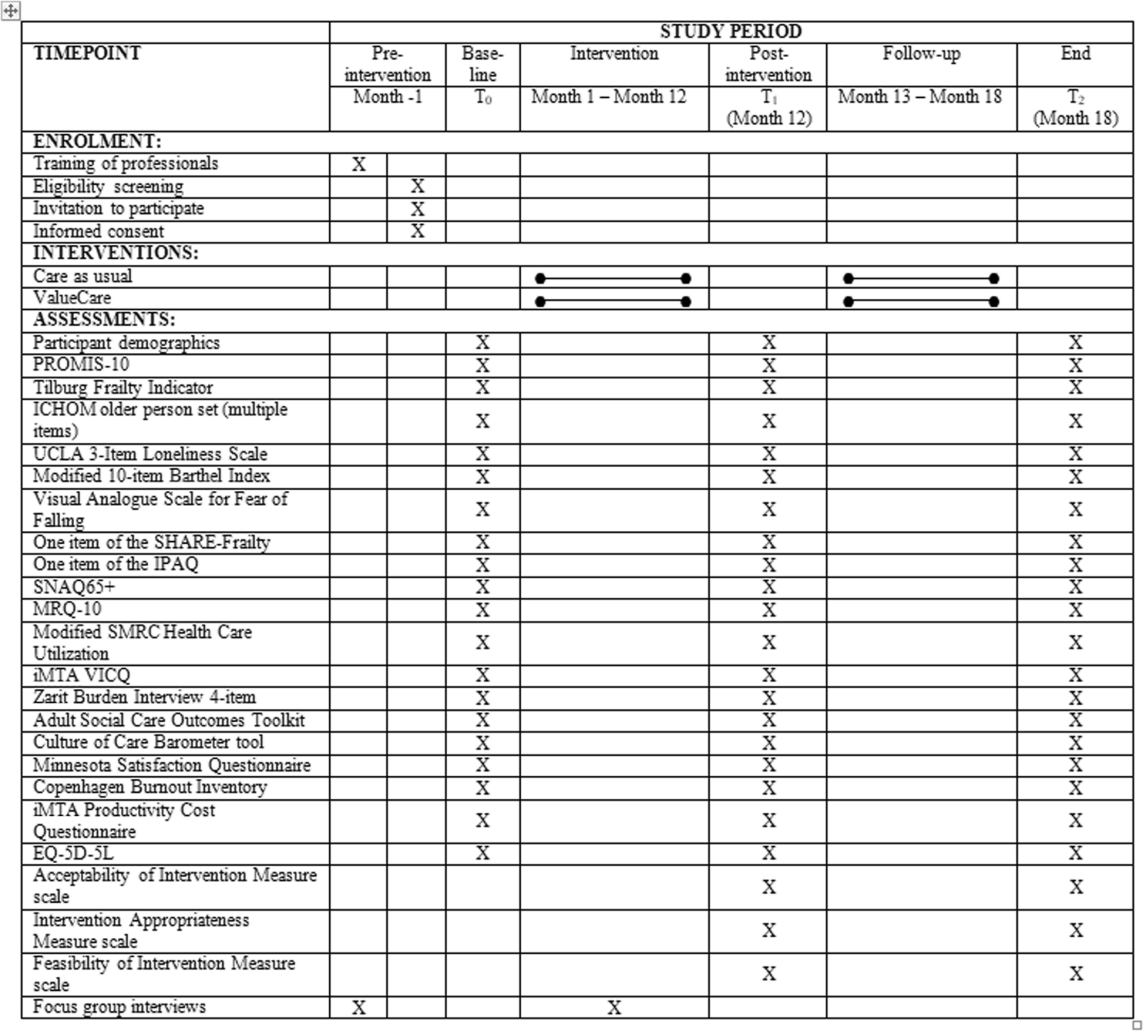
Timeline of enrolment, interventions and assessments

### Participants

The ValueCare target group consists of older people living with: (1) medical conditions and/or disabilities, (2) frailty and/or, (3) mild to moderate cognitive impairment; also their informal caregivers (e.g. relatives, friends), and health and social care practitioners will be involved in the study. Each site in the seven participating countries performs the study in accordance with the contextual and organisational factors and capacity (see Table 2).

**Table 2:** Target group per site

Site	Target group of older people
Athens (Greece)	Type II Diabetes Mellitus and hypertension as comorbidity, living independently in the community
Coimbra (Portugal)	Patients/clients with no or mild cognitive impairment, and two or more chronic conditions, and a lack of social or familiar support
Cork/Kerry (Ireland)	Older people (≥ 75 years old) with mild to moderate frailty
Rijeka (Croatia)	Patients who had a myocardial infarction, with inclusion after the clinical phase of rehabilitation
Rotterdam (the Netherlands)	Patients who had an ischemic stroke
Treviso (Italy)	Mild cognitive impairment and/or frailty, in combination with hypertension, or diabetes or cardiovascular diseases
Valencia (Spain)	Mild to moderate frailty

We aim to include 1680 *older people* (i.e. patients, clients) in total: 120 participants in the intervention group and 120 participants in the control group in each site. Study participants will be included if they: (i) are aged ≥ 65 years, (ii) have a confirmed diagnosis of the targeted chronic condition at the time of enrolment, (iii) are community-dwelling (not living in long-term care facilities) or are temporarily in a hospital or institution and are expected to be referred to their home, and (iv) are able to give informed consent.

With regard to the older people enrolled in the study, the researcher will ask the participant whether they have an informal caregiver, and will ask who is/are the most relevant formal caregivers. These informal and formal caregivers will be approached (with the permission of the participant) and invited to participate in the study. In each of the seven sites, we aim to enrol 50–70 *informal caregivers* (e.g. relatives, friends) and 30–40 *health and social care practitioners* who work with older people having the targeted condition.

### Recruitment

Study enrolment is planned to be conducted between the end of 2021 and summer 2022. Participants will be recruited with the support of health and social care providers. Physicians, nurses and other care professionals are invited to discuss the project with eligible patients who visit the hospital or care centre. In addition, health and social care providers can invite patients to participate in the study by sending a letter to introduce them to the project. Posters and brochures will also be made available throughout care facilities to support recruitment. All participants who provide informed consent and participate in the data collection at baseline are enrolled in the study.

### Intervention: the ‘ValueCare approach’

#### Co-design component in ValueCare

Prior to the start of the intervention, in ValueCare, older people, their informal caregivers, health and social care practitioners, as well as other stakeholders (e.g. policymakers, managers, ICT experts) are progressively involved in a co-design iterative process to assess the ValueCare concept and technical solution. In this regard, co-design enables patients, their caregivers and healthcare staff to reflect on their experiences of a service and to identify improvement priorities [36, 37]. Furthermore, co-design ensures the technical solution is tailored to the needs and preferences of end users regarding content and usability [38].

Each site will engage at least 40 older people, 40 informal caregivers, 20-30 health and social care practitioners, and 5–10 other stakeholders in semi-structured interviews, focus group discussions, or workshops. Qualitative analysis methods will be used to gather stakeholders’ perspectives on care experience, service provision, priorities for improvement and how digital solutions can contribute to such improvements. The co-design sessions are organised in two rounds between April 2020 and the end of 2021.

#### The ‘ValueCare approach’

Based on the E-health Enhanced Model for Chronic Care Management the ‘ValueCare approach’ will be developed and validated [39]. In order to do so, knowledge from the literature, as well as the findings from the co-design activities will be used as input. The ‘ValueCare approach’ consists of six key components: (1) health system support, (2) self-management support, (3) delivery system design, (4) clinical decision support, (5) care information systems, and (6) digital education.

#### The ‘ValueCare approach’: care pathways

Each site will apply the design of an integrated care pathway based on the ‘ValueCare approach’ for the target population. Care pathways map out the care journey an individual can expect given a certain (chronic) condition [40]. Each site designs a ValueCare pathway in its specific context based on co-design activities, the ValueCare approach and the current care pathways.

#### The ‘ValueCare approach’: outcome-based care delivery

In this project, an ‘outcome-based’ (or ‘value-based’) approach will be applied aiming to achieve better health outcomes and patient experiences. In the ‘ValueCare approach’, care professionals will measure and use the ‘outcomes’/ ‘values’ that are important for patients (clients) [41]. In all sites, in the intervention group, the ‘*value-based care approach’* will be applied to assess, discuss with patients (clients), and monitor ‘outcomes’ that are relevant to the patient (client). This is a specific application of ‘outcome-based care delivery’ developed by the International Consortium for Health Outcomes Measurements (ICHOM) [42]. It entails that a self-reported questionnaire will be administered to assess ‘outcomes’ that are relevant to the patient (client); examples are physical, mental, and overall well-being of the patients (clients). The aim of this assessment is to identify the individual care needs of the participating patients (clients) in the intervention group, and to discuss and monitor the findings with the patient (client) and their caregivers. Based on the assessment’s outcomes and detected needs, with each patient (client) a personalised care plan will be decided upon. This care plan is co-produced by the patient, (when applicable) their informal caregiver and their health or social care practitioner. The shared care plan will be periodically reviewed and can be adjusted according to the patients’ (clients’) health, wellbeing and preferences.

#### ValueCare technical solution

The ‘ValueCare approach’ will include technical solutions to support patients (clients), their informal caregivers, and their health or social care practitioners. The ValueCare technical solutions will enhance the assessment and the monitoring of the personalised care plan by a mobile health application for older people. Participants will have access to a potential range of functionalities linked to their individual care plan using a motivational and goal-setting approach, such as lifestyle coaching, disease management (e.g. medication monitoring) and care provider-participant communication. Additionally, a “Virtual Assistant” will support the accomplishment of the personal goals set by the participant and their care provider in a shared decision process. Therefore, this virtual assistant will act as a conversational agent that can interact with the participant through a chat bot following person-centredness principles and using artificial intelligence. Furthermore, wearable sensors, including fitness trackers can be added as part of the ValueCare technical solution to enhance activity monitoring of the participant. Moreover, if the participant provides consent, informal caregivers, and health and social care practitioners can have access to a web-based application, which monitors the progress of the patient (client). Participating health and social care practitioners, as well as the older people and their informal caregivers, will be invited to use the digital solutions in accordance with their roles. Additionally, capacity building activities will be provided using a ‘train the trainers’ methodology for the adoption and implementation of the ValueCare sites.

### Data collection

Data will be collected through self-reported questionnaires filled in by older people, informal caregivers and health and social care practitioners. Assistance to fill in the questionnaire will be provided by the research team if necessary. Additionally, with permission of the participant, data will be collected from clinical sources, and from the ValueCare technical solution. The general data collection instruments used are based on the Standard Set for Older Person developed by ICHOM [24, 42]. This standard set includes outcomes that matter to older people and therefore fits the purpose of ValueCare to deliver value-based care. Sites can apply particular ICHOM Standard Sets according to the specific (chronic) condition(s) of their target population. The instruments used for the outcome measures are described in the outcome measures section. Instruments for which no validated translations are available will be translated (forward and backward translations). Before starting the study, the questionnaire will be tested and validated in all sites to assure its user-friendliness in terms of appropriateness, comprehensibility and usability. Basic psychometric indicators (e.g. internal consistency) will be calculated when applicable.

#### Evaluation of health, wellbeing, quality of life, lifestyle behaviour, and health care use outcomes in older people

Table 3 describes the outcome measures used in the evaluation for older people. In addition, collected wearable data can provide information on for example number of steps taken or sitting time that can be used to enhance the self-reported data. The main outcome is the health-related quality of life (HR-QoL) score measured by the PROMIS Scale v1.2 – Global Health (PROMIS-10) representing physical health, pain, fatigue, mental health, social health, and overall health [43]. The PROMIS-10 is a domain-specific quality of life instrument that has been validated by modern psychometric methods and computerised adaptive testing to ensure greater precision and less burden [44]. Additional outcome measures include health and wellbeing outcomes, outcomes related to lifestyle behaviour, and care use. Methods and instruments have been selected because they are patient-centred, well-validated, and comprehensive measures that can be self-administered. This allows comparing our results with other studies.

**Table 3:** Effectiveness outcomes in older people

Outcome	Outcome measure(s)	Methods and instruments
Health, wellbeing and quality of life	Physical HR-QoL Mental HR-QoL	PROMIS-10 [43]
	Frailty	Tilburg Frailty Indicator [45]
	Comorbidities	ICHOM Older Person Set [42]
	Loneliness	UCLA 3-Item Loneliness Scale [46]
	Activities of daily living	Modified 10-item Barthel Index [47]
	Falls	Visual Analogue Scale for Fear of Falling [48]
Lifestyle behaviour	BMI	ICHOM Older Person Set [42]
	Smoking status	ICHOM Older Person Set [42]
	Alcohol consumption	ICHOM Older Person Set [42]
	Physical activity	One item of the SHARE-Frailty [49]
		One item of the International Physical Activity Questionnaire (IPAQ) [50]
	Nutrition and undernutrition	SNAQ65 + [51]
	Medication intake	Medication Risk Questionnaire (MRQ-10) [52]
Care use	Care utilization	Modified SMRC Health Care Utilization questionnaire [53]

#### Evaluation of wellbeing, perceived burden, and (job) satisfaction outcomes in informal caregivers, and health and social care practitioners

Table 4 summarises the effectiveness outcome measures used for informal caregivers and health and social care practitioners. Regarding indicators of wellbeing, perceived burden, and (job) satisfaction, we hypothesise more favourable results at follow-up compared to baseline measurement. Selected methods and instruments aim to provide a complete and comprehensive overview of perceived wellbeing, burden and satisfaction of participants engaged in the implementation of the ValueCare approach.

**Table 4:** Effectiveness outcomes in informal caregivers and health and social care practitioners

Outcome	Outcome measure(s)	Methods and instruments	Target group(s)
Wellbeing	Physical HR-QoL Mental HR-QoL	PROMIS-10 [43]	All
Perceived burden	Carer burden	iMTA Valuation of Informal Care Questionnaire (iVICQ) [54]	Informal caregivers
		Zarit Burden Interview 4-item [42, 55]	
	Autonomy and control	Adult Social Care Outcomes Toolkit [42, 56]	
Job satisfaction	Working conditions	Culture of Care Barometer tool [57]	Health and social care practitioners
	Satisfaction	Minnesota Satisfaction Questionnaire—Short Form [58]	
	Work-related burnout	Copenhagen Burnout Inventory [59]	

#### Evaluation of implementation outcomes in terms of acceptability, appropriateness, feasibility, fidelity, and costs

Table 5 provides the implementation outcomes and related measures for evaluating the performance of the ValueCare approach implementation across the seven sites. The implementation outcome evaluation is based on the taxonomy of implementation outcomes defined by Proctor et al. (2011) [60]. Included implementation outcomes are acceptability, appropriateness, feasibility, fidelity, and costs [60]. A mixed methods approach is used to collect implementation outcomes. This includes the 12-month self-reported follow-up questionnaires (*T*_1_), focus group interviews and data routinely collected by the ValueCare application. Focus groups will be held with older people, informal caregivers, and health and social care practitioners 12 months after implementation, and at the end of the intervention. At least 2 focus groups will be held in each site with *n* = 8–12 participants per focus group. Participants will be asked to share their experiences, for example, regarding shared-decision making, satisfaction with care, perceived fit and barriers and facilitators to implement the ValueCare approach.

**Table 5:** Implementation outcomes

Outcome	Outcome measure(s)	Methods and instruments	Target group(s)
Acceptability: willingness to receive the service offered	Enrolment rate (%)	Comparison of reported enrolment rates and targets set for the study	Older people
	Attrition/retention rate (%)	Descriptive statistics and reasons for non-consent	Older people
	Engagement	*T*_1_ follow-up questionnaire. Examples of items: engagement of patient in care plan, app functions used, cooperation between patient and care team members	All
	Perceived acceptability	4-item Acceptability of Intervention Measure (AIM) scale [61]	All
		Focus group interviews with a sample of patients, informal caregivers, and care team members	All
Appropriateness: perceived fit, relevance and compatibility of the service	Perceived fit	4-item Intervention Appropriateness Measure (IAM) scale [61]	All
		Focus group interviews with a sample of patients, informal caregivers, and care team members	All
Feasibility: extent to which a service is successfully used	Training of end users	Evaluation of training materials	All
	Perceived delivery of the intervention	4-item Feasibility of Intervention Measure (FIM) scale [61]	All
	Perceptions of barriers and facilitators	Focus group interviews with a sample of patients, informal caregivers, and care team members	All
Fidelity: extent to which the service was implemented as prescribed in the original protocol	Engagement rate (% at least one month app use)	Descriptive statistics	All
		Several items in the *T*_1_ follow-up questionnaire	
		Focus group interviews with a sample of patients, informal caregivers, and care team members	
	Dose delivered (completeness)	File analysis and *T*_1_ follow-up questionnaire: presence of care plan, app functions used, number of (digital) interactions between patients and care team	All
	Perceived quality of the delivery	Focus group interviews with a sample of patients, informal caregivers, and care team members	All
Costs: from a societal perspective	Productivity losses	iMTA Productivity Cost Questionnaire (iPCQ) [62]	Older people, informal caregivers
	Health care use	SMRC Health Care Utilization questionnaire [53]	Older people
	Quality of life	EQ-5D-5L [63]	Older people

The costs of implementing the ValueCare approach in each setting will be estimated and reported using standard procedures [64, 65]. The direct costs of using all types of health and social care services will be measured by a modified version of the SMRC Health Care Utilization questionnaire [53]. Services specific to the ValueCare implementation including training, outreach services, and time spent by care team members on elements of the program will be captured and quantified. Real cost prices will be used when unit resource prices are not available. The iMTA Valuation of Informal Care Questionnaire (iVICQ) is used to report an informal caregiver’s time spent on activities to care for a patient. Societal costs will be calculated by productivity losses for informal caregivers who perform paid labour during the study period using the friction cost method [66]. The incremental cost-effectiveness ratio (ICER) will be expressed as costs per quality-adjusted life years (QALYs) gained, based on EQ-5D-5L scores

### Power calculation

In each of the seven sites, 120 participants will be included in the intervention group and 120 participants in the control group. Assuming a 20% participant loss to follow-up between *T*_0_ and *T*_1_ (e.g. due to disability, rehousing, mortality, study withdrawal), we expect to get complete data from 672 participants in the intervention group and 672 participants in the control group of all sites at follow-up; in total *n* = 1344 study participants. We assume equal standard deviations in the intervention group and the control group, alpha of 0.05 and power of 0.80. Thus, given seven participating study sites each with an intervention group and control group, we applied a correction factor to account for the cluster design, assuming an average cluster size of 96 older citizens (1344/14) and an intra-class correlation coefficient of 0.02. For this expected overall sample size and assumptions, regarding the continuous outcome measures, a difference of 0.23 SD between the intervention and the control group can be detected at follow-up. This means that both at the European level and within each individual site, small differences regarding the outcomes in the intervention group compared to the control group can be shown [67].

### Data management and analyses

A data management plan is being developed as part of the ValueCare project and will be updated throughout the project. The document describes the data life cycle, from definition to reuse after the project. It follows a privacy-by-design approach and includes procedures for ensuring a high-quality data standard, in compliance with the FAIR principles. As the project will collect health-related data, special attention is attributed to the role of each partner in terms of controllers and processors, and to the organisational and technical measures to be put in place to ensure General Data Protection Regulation (GDPR) compliance. In addition, the risks associated to data processing will be defined in the Data Protection Impact Assessment (art. 35 GDPR) to be evaluated together with the Controllers’ Data Protection Officers. Erasmus University Medical Center is responsible for the data management, analysis and reporting.

Descriptive statistics will be used to describe participant characteristics in each site and in the total study population. Differences between *T*_0_, *T*_1_ and *T*_2_ measurements are evaluated using multilevel linear regression analyses for continuous outcome variables and multilevel logistic regression analyses for dichotomous outcome variables. We will perform subgroup analyses through formal interaction tests including those variables that are likely to influence the effect of the intervention itself, such as age, sex, living situation, education level and the baseline status of the outcome variable. Statistical analyses are repeated for each site separately. We consider a P-value of 0.05 or lower to be statistically significant.

To assess changes in implementation outcome measures from baseline (*T*_0_) to follow-up (*T*_1,_ *T*_2_) *t* tests for continuous measures and chi square for categorical variables will be used. Qualitative analysis will be performed on the focus group data. Focus groups will be digitally recorded and transcribed. The data will be managed using N-Vivo 10 software. The data will be analysed using thematic content analysis.

Using the baseline measurement as control group, a preliminary cost-effectiveness analysis will be performed from a societal and healthcare perspective. The healthcare costs per individual participant will be calculated by multiplying resource use (e.g. doctor appointments, hospital admissions) with corresponding unit prices. The results from the iPCQ are used to determine productivity losses for individual participants (lost productivity at paid work due to absenteeism and lost productivity at unpaid work). Information from the EQ-5D-5L will be used to calculate utility values.

## Discussion

This study aims to evaluate the ValueCare approach in comparison with ‘usual care’ practices in terms of benefits for the target groups (older people, their informal caregivers, and health and social care practitioners), and to evaluate implementation outcomes. Benefits of the intervention will be measured in multiple domains; for older people: health-related quality of life (HR-QoL), frailty, comorbidities, loneliness, activities of daily living, falls, BMI, smoking status, alcohol consumption, physical activity, nutrition and undernutrition, medication intake, and care utilization; for informal caregivers: health-related quality of life, caregivers’ burden, and autonomy and control; for health and social practitioners: health-related quality of life, working conditions, job satisfaction, and work-related burnout. Implementation outcomes will be measured in terms of acceptability, appropriateness, feasibility, fidelity, and costs. A pre-post controlled design is used to explore the effects of the ValueCare approach in seven European sites in Athens, Greece; Coimbra, Portugal; Cork/Kerry, Ireland; Rijeka, Croatia; Rotterdam, the Netherlands; Treviso, Italy, and Valencia, Spain.

We expect to encounter some challenges in the study. Firstly, previous research has indicated that recruiting and retaining older people with chronic conditions in research studies can be difficult due to for example reduced vision and hearing, the severity of health problems, or fatigue [68]. For that reason, the recruitment strategy seeks to encourage the participation of this population by providing a fair opportunity for them to participate and to ensure we reach our target sample size. Furthermore, capacity building activities including training sessions and regular communication with health and social care practitioners will be put in place by local study teams to reduce recruitment challenges and increase the adherence to the study. Furthermore, it is possible that some elements of the technical solution may not be used by older people, family members or professionals due to the lack of interest or an unfriendly interface design [69]. To encounter these challenges, the research team developed the intervention implementing a key co-design process of the ValueCare approach and technology solution. Moreover, training activities are expected to facilitate the implementation of the intervention and the use of the new technology, increasing the adherence to the ValueCare intervention and use of the technical solutions by the target groups [37].

Moreover, this study has several strengths which are important to stress. First of all, the ValueCare project addresses challenges of fragmentation in providing integrated care for a growing number of older people with multimorbidity, frailty, and mild to moderate cognitive impairment. Second, the study combines the evaluation of effectiveness outcome measures and the process. This comprehensive approach to evaluation will help to understand the complexity of the interactions between many contextual factors, and therefore contributes to reducing the research-to-practice gap [70]. Third, this study explores the effects of the ValueCare approach among diverse older adult populations in seven different European settings which generates contextual information on its generalisability and feasibility. By utilising a uniformed questionnaire and measurements, including the ICHOM Standard Set for Older Person, a cohesive evaluation will be applied. Fourth, the ValueCare technical solution will be co-designed with end users to ensure the solution serves their needs and preferences [71]. The use of ICT can increase patient empowerment by allowing users to have insight in their health data [72].

In summary, the results of this study will provide evidence on the benefits of an innovative and value-based integrated care approach that could potentially support the ‘Quadruple Aim’ regarding care for older people with multimorbidity, frailty, and mild to moderate cognitive impairment. By developing a model of care following the principles of value-based health care and integrating health and social care, supported by appropriate technical solutions within current practices across seven European countries, this study can contribute to new ways of providing person-centred and value-based integrated care supported by ICT solutions to older people.

## Data Availability

The datasets used during the study are available on reasonable request by contacting the corresponding author.

## Abbreviations

BMI: Body Mass Index
EQ-5D-5L: 5-Level EQ-5D
GDPR: General Data Protection Regulation
GP: General practitioner
HR-QoL: Health-related quality of life
ICER: Incremental cost-effectiveness ratio
ICHOM: International Consortium for Health Outcomes Measurements
ICT: Information and Communication Technology
iMTA: Institute for Medical Technology Assessment
iVICQ: IMTA Valuation of Informal Care Questionnaire
iPCQ: Productivity Cost Questionnaire
PROMIS: Patient-Reported Outcomes Measurement Information System
QALYs: Quality-adjusted life years
SMRC: Self-management resource center
ValueCare: Value-based methodology for integrated care supported by ICT
